# Recent advances in threshold-dependent gene drives for mosquitoes

**DOI:** 10.1042/BST20180076

**Published:** 2018-09-06

**Authors:** Philip T. Leftwich, Matthew P. Edgington, Tim Harvey-Samuel, Leonela Z. Carabajal Paladino, Victoria C. Norman, Luke Alphey

**Affiliations:** 1 The Pirbright Institute, Pirbright, Woking, Surrey, U.K.; 2 Department of Zoology, University of Oxford, Oxford, U.K.

**Keywords:** *Aedes aegypti*, frequency dependent, gene drive, genetic modification, underdominance, *Wolbachia*

## Abstract

Mosquito-borne diseases, such as malaria, dengue and chikungunya, cause morbidity and mortality around the world. Recent advances in gene drives have produced control methods that could theoretically modify all populations of a disease vector, from a single release, making whole species less able to transmit pathogens. This ability has caused both excitement, at the prospect of global eradication of mosquito-borne diseases, and concern around safeguards. Drive mechanisms that require individuals to be released at high frequency before genes will spread can therefore be desirable as they are potentially localised and reversible. These include underdominance-based strategies and use of the reproductive parasite *Wolbachia*. Here, we review recent advances in practical applications and mathematical analyses of these threshold-dependent gene drives with a focus on implementation in *Aedes aegypti*, highlighting their mechanisms and the role of fitness costs on introduction frequencies. Drawing on the parallels between these systems offers useful insights into practical, controlled application of localised drives, and allows us to assess the requirements needed for gene drive reversal.

## Introduction

A gene drive can be described as any system in which genes enhance their own representation in a sexually reproducing population, beyond that of Mendelian transmission, even if the inserted genetic element reduces the overall fitness of the organism [1–3]. The ability to spread genes through a population that do not benefit the individuals carrying them has applications for the control of disease vectors, for example by spreading genes that reduce the transmission efficiency of arboviruses by mosquito hosts [4–7]. Inherent in most gene drive systems is the requirement for individuals to be released above a certain threshold frequency before they will drive, though for some systems this will be extremely low (and even then only when considering the requirement to overcome stochastic effects at extremely low-frequency introductions) — this threshold being determined as a combination of the action of the system and its fitness load [1].

Gene drives based on homing endonucleases such as CRISPR/Cas9 are likely to produce highly invasive systems [8,9]; as these will require extremely low-frequency releases, unless fitness costs are very high, or resistant mutations arise [10,11]. As a result of this high invasiveness, homing drives have the capacity to be highly effective, but simultaneously problematic as they will inevitably cross geo-political borders and produce potentially irreversible changes in population genetics [8,9]. There is, therefore, still a requirement for alternative gene drives that are capable of spreading and maintaining a selected cargo (e.g. imparting disease refractoriness) in a localised and reversible manner (though see alternative proposals to localise/reverse CRISPR/Cas9 systems[12–14]). Promising alternatives come from the development of systems that generate drive by imposing high fitness costs during outbreeding, such as artificial underdominance (UD) or using the reproductive parasite *Wolbachia* [15–19]. The distinction between these and homing drives is that they require much higher release numbers in order to establish as a self-sustaining gene drive, and are actively driven out of a population if released below that invasion threshold. This approach has the capacity to be spatially self-limiting [20], and reversible through the re-introduction of wild-type organisms [16,21,22].

We describe below recent empirical and modelling advances in threshold-dependent gene drives. We compare the potential invasiveness of differing approaches at different fitness loads. Finally, as many of these systems are proposed as ‘reversible,’ we discuss the requirements for attempting ‘drive-out’ once such gene drives have already reached high frequency.

## Underdominance

**Figure 1.**
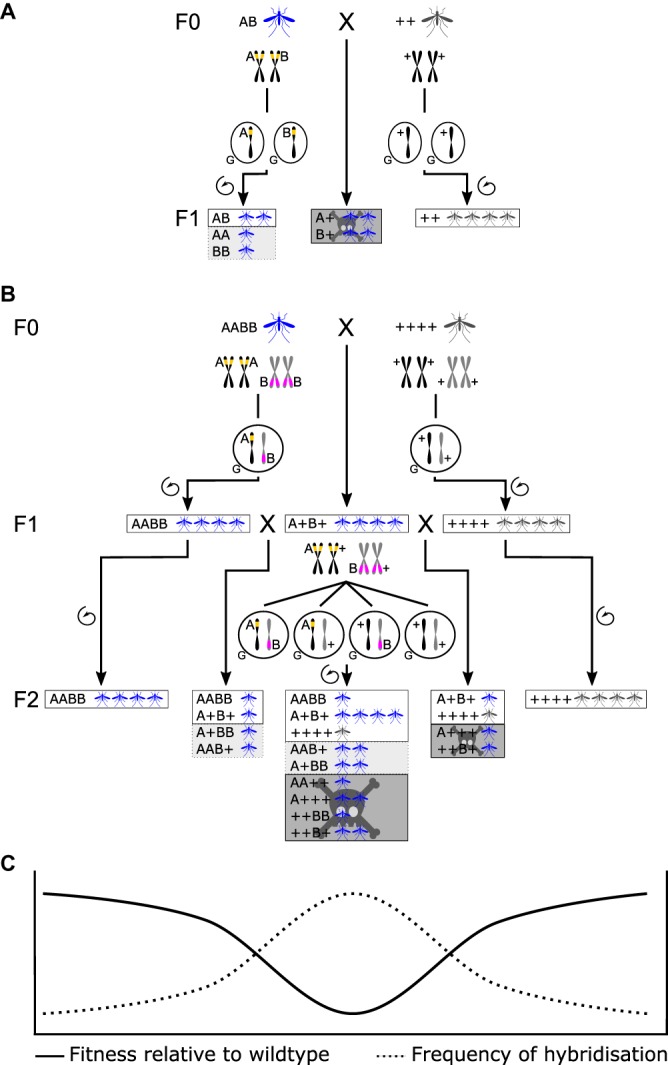
Mating outcomes through inbreeding and hybridisation in one and two locus underdominance. (**A**) One-locus UD A&B are mutually suppressing dominant genetic elements at the same locus. ‘Parental’ lines are either heterozygous (in blue) for both A and B alleles at the same loci (AB) or wildtype (in red) (++). Crosses between transgenic and wildtype strains (middle stream) produce F_1_ genotypes which carry unsuppressed lethals (A+ or B+) and are not viable. All inbreeding of wildtype (right stream) produces fully viable offspring (++). All inbreeding of AB strains (left stream) produces 50% viable offspring (AB) and 50% offspring that are homozygous at the same locus (AA or BB) and are inviable, and are highlighted in light grey (see next). For haploinsufficient RNAi, there is only one modified genetic element at the locus, ‘parental’ lines are AA only and no inviable genotypes are produced by inbreeding. In this scenario, the light grey highlighted box is viable. (**B**) Two-locus UD A&B are mutually suppressing unlinked dominant genetic elements, ‘parental’ lines are either homozygous for both A and B (AA,BB) or wildtype (++,++). All inbreeding of either wildtype or AA,BB strains (right and left streams, respectively) produces fully viable homozygous offspring. F_1_ hybrids between these strains (A+,B+) (middle stream) are also viable; however, some of the F2 progeny are non-viable. F_1_ hybrids therefore have reduced fitness compared with either parental homozygote. As all F_1_ hybrids are viable, here we illustrate crosses of these F_1_ hybrids to each other and both parental strains. Genotypes carrying unsuppressed lethals are highlighted in dark grey. If a single copy of a suppressor were insufficient to suppress two copies of a lethal, then these genotypes would also be inviable, these genotypes are highlighted in light grey. (**C**) The relationship between levels of hybridsation and fitness at the population level for interbreeding between modified populations and wildtype. Fitness is highest when individuals from either population inbreed, as the frequency of hybridisation increases, the relative fitness of the population falls.

**Figure 2.**
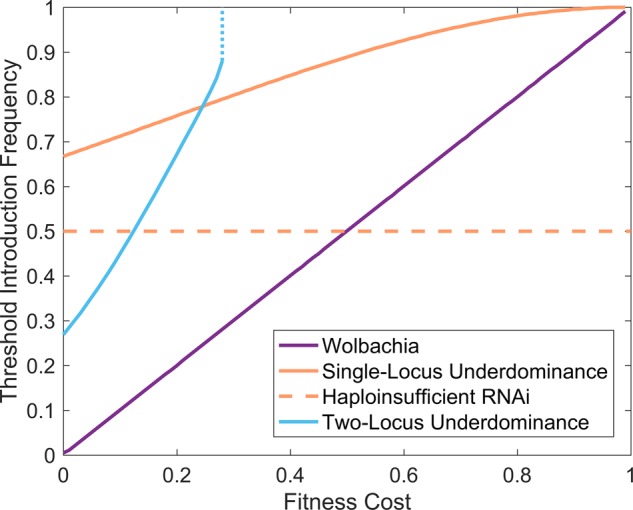
Comparison of predicted introduction threshold frequencies for *Wolbachia*, single-locus UD, haploinsufficient RNAi and two-locus UD systems. For each fitness cost parameter (relative to wildtype), a population genetics mathematical model is repeatedly simulated for different introduction frequencies with the first (lowest) frequency giving successful introgression being output to form the threshold lines seen here. For two-locus UD, the dotted portion of the line indicates a maximum fitness cost beyond which introgression cannot be achieved. In the case of haploinsufficient RNAi, it is assumed that fitness costs affect only heterozygotes, i.e. wildtype and homozygotes are of equal fitness. The four mathematical models used here are adapted from those of Marshall and Hay [31] except that for haploinsufficient RNAi which is from Reeves et al. [29].

The principle of using UD as a mechanism for driving desirable genes into mosquito populations has been around for several decades [17,23]. UD is a genetic property classically defined as the condition where, at a single locus, the fitness of heterozygotes is lower than that of either corresponding homozygote; generating this effect has been proposed as achievable through either chromosomal translocations or mutually suppressing transgenic toxin-antidote elements (though none have as yet been developed in exactly the latter format; Figure 1) [16,17,23,24]. Single-locus UD establishes a population level, frequency-dependent gene drive, with the likelihood of an UD gene drive establishing total wild-type replacement depending upon the initial frequency of introduction and the fitness of the introduced homozygotes relative to wildtype, but does not require that the introduced homozygotes have an equal fitness to wildtype [23] (Figure 2). The term UD is also applied to two-locus systems where hybrids have lower fitness than either true-breeding parental strain. In engineered two-locus UD, designs are based on a pair of mutually suppressing dominant lethal genes. Two genetic elements are located at independently segregating loci, individuals are viable when they inherit at least one copy of each allele (Figure 1) [17,20]. First generation hybrids are viable, but suffer a greatly reduced fitness at reproduction when the constructs segregate. This first generation viability allows introgression of both genetic elements into a population at a lower introduction threshold than for single-locus UD, assuming low fitness costs (Figure 2); however, fixation of genetic elements is not guaranteed — at even moderate fitness costs a stable equilibrium may instead be attained with a balance of homozygotes and heterozygotes in the population. Alternatively, though higher release numbers are needed, drive can be achieved by undertaking single-sex (male) releases, as a trade-off against releasing biting females — only female mosquitoes bite — the mass-release of single sexes having shown to be viable in other insect control measures such as SIT or RIDL [25–27].

In recent years, there have been rapid advances in tools available to molecular biologists, and this has led to several innovative new approaches towards establishing UD-based gene drives and fresh interest in their potential as stand-alone or complementary technologies to homing drives in mosquitoes [28]. We review some of the most recent advances in UD below (for summary, see Table 1).

**Table 1:** Overview comparison of the gene drive systems explored in this manuscript Note that this table is for broad comparison purposes only and individual parameters (e.g. invasion thresholds) for systems grouped together (e.g. various 2-locus systems) will differ from one another. Equally, for a single system (e.g. unidirectional *Wolbachia*), characteristics will differ depending on the specifics of that system (e.g. fitness costs/maternal transmission rates and penetrance of cytoplasmic incompatibility).

	*Wolbachia*		Engineered UD	
	Bidirectional	Unidirectional	One-locus^1^	Two-locus^2^
Status of modified vector	Non-GM	Non-GM	GM	GM
Intended outcome of drive deployment	Replacement	Replacement	Replacement/suppression (dependent on cargo gene)	Replacement/suppression (dependent on cargo gene)
Method of deployment	Requires bisex release	Requires bisex or female-only release	Requires bisex release (UD^MEL^ can be established with male-only release)	Can be established with male-only releases
Relative introduction allele frequency threshold (assuming equal fitness with existing wild population)	High (>0.5)	Low (>0) Current field strains estimated at ∼0.3	High (>0.5)	Low (<0.5)
Relative invasiveness	Low Assuming recipient non-target population has same *Wolbachia* infection as target population	High Assuming recipient non-target population has no existing *Wolbachia* infection	Low	High
Relative ‘drive-out’ ability	High Requires wild-type bisex release	Low Requires wild-type bisex release	High Requires wild-type bisex release (UD^MEL^ can use single-sex female release)	Low Can be achieved by releasing only wild-type, non-biting, males
Current development status	Proposed [32]	Field testing in *A. aegypti*	Laboratory proof-of-principle in *D. melanogaster* Laboratory development in *Culex quinquefasciatus*	Laboratory proof-of-principle in *D. melanogaster* Laboratory development in *A. aegypti*

1 Includes haploinsufficient, (1-locus) toxin-antidote (proposed but not yet developed), PTA overexpression, and (1-locus) UD^MEL^ based systems.

2 Includes (2-locus) toxin-antidote (proposed but not yet developed), (2-locus UD^MEL^) and reciprocal chromosomal translocations based systems.

KEY: Drive outcome — goal of deploying drive. Either replacement of wild population with modified, less harmful population or suppression of wild population density. For engineered UD systems, outcome will depend on cargo gene tightly linked to other drive components. Bisex release: Release into the wild of individuals from both sexes, usually in roughly equal number. Introduction allele frequency threshold: The frequency the released modification must reach in the wild population before the drive will then begin to spread. Above this frequency, the drive conveys a population-level fitness advantage. Invasiveness: Propensity for a released drive to begin spreading in populations other than the one into which it was released. Note that these are relative invasiveness levels and all these systems are regarded as relatively non-invasive compared with other systems such as homing drives. Drive-out ability: The relative ease with which a population where the released modification has spread to fixation can be returned to its original non-modified state through the release of wild-type (non-modified) individuals. Note that this is the inverse of the introduction threshold/invasiveness of the system.

### Haploinsufficient RNAi

The use of haploinsufficient genes as a system to generate artificial UD has been developed as a proof-of-principle in *Drosophila melanogaster* [29]. Expression of the ribosomal protein gene *RpL14* was knocked down by an RNAi transgene. Many ribosomal protein genes are haploinsufficient in *D. melanogaster*; individuals with only one functional copy have severe defects including delayed development and small size. The RNAi component suppresses the endogenous *RpL14* genes, while transgenic homozygotes are rescued from the knockdown effect by carrying two copies of a synthetic RNAi-resistant ‘rescue’ version of the same gene *RpL14*. However, hybrids have only one copy and therefore have greatly reduced fitness — an underdominant system (Figure 1). This system requires no particular sex or tissue specificity to function, and has potential for implementation in a wide variety of organisms; ribosomal genes being highly conserved in function across fungi, plants, invertebrates and vertebrates. Such systems are predicted to have high invasion thresholds, therefore needing a high release rate to establish but being relatively controllable and reversible (Figure 2). Caged population trials of this system were observed to reach fixation of the genetic elements when initial releases were made above a single release frequency of 0.61.

### Maternal effects

The maternal-effect lethal underdominance (UD^MEL^) system [21], utilises two constructs, each expressing different maternally deposited micro-RNAs that act as embryonic toxins, with an embryonically expressed rescue gene, using components similar to a synthetic *Medea* design [30]. However, here each rescue gene is attached to an opposite toxin, so toxin A is linked with rescue B and *vice versa*. Survival of embryos is contingent on inheriting both constructs simultaneously. This system can be built to function as either a single- or two-locus UD system; unlike most proposed engineered UD systems, toxins are only expressed/deposited by mothers, not fathers, such that transgenic heterozygotes are always viable when the transgenic parent is male. This allows for the potential of ‘male-only’ releases in both single and two-locus forms.

In a single-locus design, the two constructs are arranged at the same locus on homologous chromosomes and only trans-heterozygotes (carrying one copy of each construct) are viable (crosses between trans-heterozygotes will produce 50% viable trans-heterozygote offspring, and 50% non-viable homozygotes). In a two-locus system, individuals would need to be made homozygous for each genetic element at independently segregating loci.

Cage experiments demonstrated the ability to drive to fixation when male-only release percentages as a fraction of the total population were greater than 50% [21].

### Reciprocal translocations

Buchman et al. [22] generated homozygous chromosomal translocations between chromosome 2 and 3 in *D. melanogaster* using two previously inserted transgenes carrying rare base cutters inserted at these different loci. This would function as a two-locus UD system as F_1_ hybrids are viable. Crosses between heterozygous chromosome translocated individuals and wildtype, generated 50% non-viable individuals (resulting from aneuploidy, non-balanced chromosomes), and 50% orthoploid (balanced chromosomes, half of these wildtype, the others balanced translocation heterozygotes bearing the reciprocal translocations). Caged population trials demonstrated that the drive system spread to fixation at introduction frequencies >50% (60, 70 and 80% tested), and drop to extinction at frequencies <50% (20, 30, 40% tested). The authors discussed that rates of spread to fixation and elimination were, respectively, slower and faster than predicted indicating unexplained fitness costs in their system. Limitations inherent to this system come from the rather unpredictable nature of fitness costs associated with chromosome translocations; multiple rounds of trial and error may be required before a strain with suitable invasion potential can be developed.

### CRISPRa

CRISPR technology has been readily applied towards the development of highly invasive gene drive systems as described above [33–35]. However, recent efforts have also been made towards developing Cas9 for less invasive UD gene drives [36]. Engineered CRISPRa (deactivated Cas9 with a fused transactivation domain [37–39]) allows a modified Cas9 to produce dominant-lethal ectopic/overexpression of endogenous genes. Lethality is prevented in the homozygous transgenic line by recoding both copies of the target promoter region such that it is no longer recognised by the CRISPRa complex. This system has promise as a drive system, in tested organisms (yeast and fruitflies) [36,40] there are a large number of promoter regions that readily respond to CRISPRa [41], resulting in a wide array of potential targets. However, evidence so far indicates that fine-tuning activation against basal toxicity, in order to make a viable transgenic strain for gene drive, is less straightforward. CRISPRa in *D. melanogaster* showed only partial efficacy — as they could not be readily assembled into a homozygous viable line in order for a drive to be tested. Those lines where the CRISPRa transgenes were homozygous viable did not show complete lethality when crossed to wildtype, slowing the potential spread of this drive system [40].

## Wolbachia

*Wolbachia*, a diverse group of maternally inherited intracellular bacteria, are present in many species of arthropods [42–44]. *Wolbachia* manifest a wide variety of different driving phenotypes such as feminising, parthenogenesis and cytoplasmic incompatibility (CI), depending on the strain of *Wolbachia* and the species infected [45–47]. CI drives *Wolbachia* infection as uninfected females have a reproductive disadvantage relative to infected females, caused by (and in proportion to the frequency of) infected males — these produce viable embryos with infected females but inviable with uninfected females. Combined with the maternal inheritance of *Wolbachia*, this results in an increase in their frequency, and any desirable traits associated with them, over multiple generations. *Wolbachia* are thus a gene drive system, with a much lower invasion threshold than predicted for UD methods (Figure 1).

*Aedes aegypti* populations are not naturally infected with *Wolbachia*, but when this is achieved they demonstrated significant pathogen blocking of viruses such as dengue, Zika and chikungunya, at least for certain strains of *Wolbachia* and through unknown mechanisms [4,48,49]. *A. aegypti* strains artificially infected with *w*Mel or *w*MelPop strains from *D. melanogaster* have been used in field trials [50–53]; to date, *Wolbachia* is the only gene drive system that has been used in open release. *w*MelPop demonstrated almost complete blocking of transmission of dengue viruses but also high fitness costs and failed to establish in target field populations [52]. *w*Mel shows partial blocking of dengue viruses in the laboratory (but see [54,55]) with lower fitness costs and in trials in Australia established and slowly spread from two of three release sites [53,56] (Table 1.)

Efforts to improve the fitness of *Wolbachia* strains in *A. aegypti* are currently underway, with the introduction of *Wolbachia* from other species such as *Drosophila simulans* (*w*Au) and *Aedes albopictus* (*w*AlbA/*w*AlbB) [57]. In the laboratory, *w*Au produced significantly better pathogen blocking than *w*Mel, but does not produce CI and is therefore incapable of spreading as a drive. Proof-of-principle research has been generated with ‘superinfections’ (multiple strains of *Wolbachia* combined in a single host), to combine the most desirable traits of individual strains, and may be a route to improvement of this system [58–60].

## Modelling of invasion potential

Modelling has been an essential tool in the successful development of genetic control measures both before and after deployment. Probably due to an increasing focus on CRISPR-based systems, there have been relatively few modelling studies for UD systems in recent years, nonetheless several key advances have been made. One recent study looked at the risk of ‘escape’ or introgression of drive systems into non-intended neighbouring populations. This showed that two-locus systems require high rates of migration (e.g. ∼5% exchanged per generation when transgene homozygosity confers a 5% fitness cost) in order to spread to high frequency in neighbouring populations, and were unable to find conditions where a one-locus system might spread [20]. The study of two-locus gene drives has also been broadened to consider a range of different genetic characteristics (e.g. female-specific lethal genes) and release scenarios (e.g. single versus multiple and male-only versus bisex releases) [61]. Laruson and Reed [62] showed that meta-populations with small numbers of migration routes between sub-populations (e.g. a linear arrangement) have a lower effective migration rate than those with many migration routes. Thus, underdominant genetic mutations are less likely to spread through a meta-population with fewer migration routes between sub-populations. This may prove an important consideration when designing and deploying UD-based gene drive systems in specific contexts.

Probably due to the field release status of *Wolbachia*-infected mosquitoes in Australia, there have been several recent studies modelling *w*Mel. One major focus of this work has been exploring factors relating to geographic spread. Models indicated that strong density-dependent larval competition can decrease female fecundity and increase larval development time, slowing the spread of *Wolbachia* when released into a wild population [63,64]. Turelli & Barton [65] found that releases must exceed a critical size (i.e. cover a large enough geographic area) to spread and that this geographic spread would be slower than expected from observations of natural *Wolbachia* invasions in other species. The presence of a critical release area appears to be supported by data from releases in Queensland, Australia [53].

In light of the current field use, many studies have sought to predict the likely epidemiological impact of successful *Wolbachia* releases on dengue prevalence in a range of scenarios, including non-endemic settings (imported cases) and areas with more than one circulating serotype [66–69]. Results in these studies suggest that whilst *Wolbachia* releases are likely to significantly reduce the number of dengue infections, at least in low transmission settings, they are unlikely to completely eliminate them.

## Drive-out

An often cited but poorly described aspect of high threshold drive system theory is that, unlike homing drives, it is possible to reverse genetically modified populations to wildtype through the release of unmodified individuals [22,62,70] (but see [21]), a mechanism which could be highly desirable should the application of any of these technologies have unintended impacts. In these systems, population replacement could be achieved by releasing wild-type individuals until they reach a frequency in which the drive system imposes a greater outbreeding cost on transgenics than wildtypes, resulting in ‘drive-out’ of the genetic elements.

The frequency of wild-type releases required to establish ‘drive-out’ from a modified population at fixation should function as a direct inverse of the threshold required for the genetic elements to invade, and could potentially require the release of large numbers of wild-type males and females in order to achieve this aim. Practical concerns may exist around the prospect of a ‘reversible system’ that requires the mass-release of additional disease vectors, and the ability to induce reversal through ‘male-only’ releases would therefore be desirable. Of the UD systems described above, the single-locus ones (haploinsufficient RNAi, single-locus UD^MEL^, CRISPRa) cannot be reversed without the release of wild-type males and females (or in UD^MEL^ just females) — as hybridisation is lethal, single-sex releases would produce no viable offspring, though released wild-type males could instead be used to induce population reduction. In a two-locus UD system (reciprocal translocations, two-locus UD^MEL^), the threshold frequency required for drive-out is more difficult to attain as this system has a greater invasion potential (though a stable equilibrium below fixation is possible here, see above). As heterozygotes are viable, it is possible to achieve drive reversal through single-sex, male-only releases. Reversal would be slower than with a bisex release, but would offset damage by not releasing additional biting females.

Reversal of *Wolbachia* systems presents unique difficulties due to the requirement for release of extremely large numbers of wild-type males and females, far in excess of that needed for either UD system as described above. *Wolbachia* infections can be replaced with other, incompatible *Wolbachia* infections, but it appears extremely difficult to get a large infected population back to an uninfected state.

## Challenges and future perspectives

Threshold-dependent gene drives have many advantages that make them suitable for localised population modification, including higher invasion thresholds, lower risk of escape, and capacity for reversal or ‘drive-out’. Previous applied use of sterile males — SIT and RIDL and ongoing trials with *Wolbachia* — demonstrate that continuous or mass releases are achievable at much higher numbers than required for any of these gene drive systems and can produce significant impact on vector populations [51,52]. The ongoing field testing of *Wolbachia-*based gene drives in *A. aegypti* demonstrates potential for field release and establishment of a gene drive system, in spite of relatively high fitness costs. Looking forward, development of alternative strains may improve the performance of the system. In comparison, UD gene drives are markedly less developed, with no field trials or established systems in *Aedes*, but many promising laboratory proof-of principles in *Drosophila* [22,23,30,40]. Invasion thresholds for UD systems are higher than those predicted for *Wolbachia* at equal fitness, making the system more local, controllable and reversible, at the cost of needing somewhat higher initial release numbers (Figure 2).

Localised gene drives represent a powerful complementary tool to more invasive homing drives, and applied separately or together provide a suite of powerful tools towards reducing insect-borne infectious disease. Theoretical underpinnings are strong, and with active experimental work in several laboratories, looking forward it is likely that there will be a range of potential gene drive systems that can be employed on a case-by-case basis within communities and countries depending on requirements.

## Abbreviations

CI: cytoplasmic incompatibility
UD: underdominance
UD^MEL^: maternal-effect lethal underdominance
